# Individual differences in the effects of midazolam on anxiety-like behavior, learning, reward, and choice behavior in male mice

**DOI:** 10.3389/fpsyt.2023.1122568

**Published:** 2023-03-03

**Authors:** Caio Jovita-Farias, Meagan E. Follett, Behaim C. Dias-Junior, Yasmim A. Serra, Natali D. Kisaki, Thaísa Barros-Santos, Nailton M. S. de Jesus, Isa R. S. Rodrigues, Larissa E. L. Macedo, Elena L. A. Malpezzi-Marinho, Alexandre J. Oliveira-Lima, Eduardo Ary Villela Marinho, James K. Rowlett, Lais F. Berro

**Affiliations:** ^1^Department of Health Sciences, Universidade Estadual de Santa Cruz, Ilhéus, BA, Brazil; ^2^Department of Psychiatry and Human Behavior, University of Mississippi Medical Center, Jackson, MS, United States; ^3^Department of Biological Sciences, Universidade Estadual de Santa Cruz, Ilhéus, BA, Brazil

**Keywords:** benzodiazepine, midazolam, elevated plus maze, self-administration, conditioned place preference, mice

## Abstract

**Introduction:**

The aim of the present study was to investigate the behavioral effects of the benzodiazepine midazolam in male mice, in models of anxiolysis, learning, and abuse-related effects.

**Methods:**

In a first set of experiments, male Swiss mice were submitted to the training session of a discriminative avoidance (DA) task on the elevated plus maze to evaluate anxiety-like behavior and learning after vehicle or midazolam (1, 2 or 5 mg/kg, i.g.) administration. The same animals were submitted to a conditioned place preference (CPP) protocol with midazolam (1, 2 or 5 mg/kg, i.g.). In a second experiment, outbred (Swiss) and inbred (C57BL/6) male mice were submitted to a two-bottle choice (TBC) oral midazolam drinking procedure. Animals were exposed to one sucrose bottle and one midazolam (0.008, 0.016 or 0.032 mg/ml) plus sucrose bottle.

**Results:**

Midazolam (1 and 2 mg/kg) induced anxiolytic-like effects, and all doses of midazolam prevented animals from learning to avoid the aversive closed arm during the DA training session. Assessment of midazolam reward *via* the CPP procedure and choice *via* the TBC procedure showed notable variability. A 2-step cluster analysis for the CPP data showed that midazolam data were well-fitted to 2 separate clusters (preference vs. aversion), albeit with the majority of mice showing preference (75%). Correlational and regression analyses showed no relationship between midazolam reward and anxiolytic-like effects (time spent in the open arms in the DA test) or learning/memory. Two-step cluster analysis of the TBC data also demonstrated that, regardless of strain, mice overall fell into two clusters identified as midazolam-preferring or midazolam-avoiding groups. Both midazolam preference and avoidance were concentration-dependent in a subset of mice.

**Discussion:**

Our findings show that midazolam preference is a multifactorial behavior, and is not dependent solely on the emergence of therapeutic (anxiolytic-like) effects, learning impairments, or on genetic factors (inbred vs. outbred animals).

## 1. Introduction

Benzodiazepines are among the most widely prescribed psychiatric medications, with more than 8% of the adult U.S. population reporting benzodiazepine use (1). This widespread use is partially driven by benzodiazepine prescriptions for one of their many therapeutic uses, predominantly anxiety and sleep disorders. However, benzodiazepine misuse also has increased in recent years, with nearly 20% of individuals who use benzodiazepines reporting misuse in the U.S. (2, 3). This has prompted a growing public health concern, particularly due to increasing rates of benzodiazepine-related overdose deaths and emergency department visits in recent years (4).

Decades of research have helped elucidate the pharmacological mechanisms underlying the behavioral effects of benzodiazepines (5). However, many questions still remain unanswered regarding their abuse-related behavioral effects, particularly due to inconsistencies in the literature. The positive reinforcing effects of benzodiazepines have been shown more consistently in studies using intravenous drug self-administration (5, 6). On the other hand, many pre-clinical self-administration studies using the oral route showed no or low reinforcing effects of benzodiazepines (7–9). Similarly, benzodiazepines have been shown to exert rewarding effects in the conditioned place preference (CPP) model in some studies (10–12), but not others (13, 14). This discrepancy is particularly relevant given that studies show that benzodiazepines can increase (15, 16), decrease (17, 18), or even not alter (19, 20) extracellular dopamine brain levels, depending on the study or protocol.

In addition to these inconsistencies, the relationship between the anxiety-decreasing (anxiolytic) effects and the abuse potential of benzodiazepines remains poorly understood. Anxiety is a risk factor for sedative, hypnotic, or anxiolytic use disorder (21), and studies have shown that greater anxiety sensitivity is associated with increased rates of non-medical benzodiazepine use (22, 23). Epidemiological studies also show that anxiety is associated with higher rates of benzodiazepine misuse and use disorder [for review see (4)]. However, whether experiencing an anxiolytic effect is associated with and/or necessary for the emergence of the positive reinforcing effects of benzodiazepines remains unknown.

The aim of the present study was to investigate the behavioral effects of the benzodiazepine midazolam in male mice, with a focus on its rewarding effects and self-administration. The rewarding effects of midazolam were evaluated using CPP, and were compared with the anxiolytic effects of this drug using an elevated plus maze-discriminative avoidance task (24). Self-administration of midazolam was evaluated using a two-bottle choice (TBC) model. Inbred and outbred mice were used in the TBC model to investigate potential broad genetic determinants of midazolam preference/avoidance.

## 2. Methods

### 2.1. Animals

Three-month-old Swiss male mice from the breeding colony of Universidade Estadual de Santa Cruz (UESC) and 3-month-old C57BL/6 male mice obtained from Harlan/Envigo were used. The first set of experiments (Swiss mice; discriminative avoidance task and CPP) was performed at UESC, and animals were group housed (8 per cage) in polypropylene cages (41 × 34 × 16.5 cm). Rodent chow (Nuvilab, Quimtia SA, Colombo, PR, Brazil) and water were available *ad libitum* throughout the experiments. The TBC experiments were performed at UESC (Swiss mice) and at the University of Mississippi Medical Center (UMMC, C57BL/6 mice), and subjects were individually housed in polypropylene caging (30 × 19 × 13 cm) with food available *ad libitum* and fluids restricted to those available within the context of the experiment 24 h per day. All animals were maintained under controlled temperature (22–23°C) and light (12 h light, 12 h dark; lights on at 06h45 at UESC and at 07h00 at UMMC) conditions.

Animals were maintained according to the National Institutes of Health Guide for the Care and Use of Laboratory Animals (8th Edition, revised 2011) and in accordance with the Brazilian Law for Procedures for Animal Scientific Use (#11794/2008). The Institutional Animal Care and Use Committees of UESC (protocol #006/2017) and UMMC (protocol #1395) approved the experimental procedures.

### 2.2. Drugs

For the discriminative avoidance and CPP experiments, midazolam (Roche^®^) was diluted in sterile saline, and administered intragastrically (gavage) at a volume of 10 ml/kg. For the TBC experiments, midazolam (Roche^®^) was diluted in a 4% sucrose solution in water to various concentrations (0.008–0.032 mg/mL).

### 2.3. Elevated plus maze-discriminative avoidance task

The elevated plus maze-discriminative avoidance task model was developed to allow for the investigation of several behaviors and behavioral effects of drugs in the same model. This model allows for the simultaneous investigation of learning/memory, anxiety-like behavior and locomotor activity (24–26). The elevated plus maze-discriminative avoidance task has been validated with the use of several different drug classes, including anxiolytic drugs such as benzodiazepines (24, 27–29) and ethanol (30, 31), stimulants (24, 26, 32–34) and opioids (35).

The behavioral sessions are performed in a modified elevated plus maze, in which the animal explores two adjacent closed arms (28.5 x 7 x 18.5 cm), one of which is aversive (aversive stimuli: 100 watts light and 80 dB noise when the animal enters the arm), and two adjacent open arms (28.5 x 7 cm) lacking the light and noise stimuli. For this experiment, a test session is performed 24 h after a training session. During the 10-min training session, animals are placed individually in the center of the apparatus with free access to the 4 arms. The aversive stimuli are activated each time the animal entered the aversive closed arm, and were interrupted when the animal leaves this compartment. The test session lasted 3 min, during which the animal was again placed in the center of the apparatus. However, during the test session the aversive stimuli were not activated when the animal entered the aversive closed arm, although the inactive lamp was still present over the closed aversive arm as an environmental cue.

All experimental sessions were filmed for later quantification of the time spent in each of the arms of the device (aversive closed arm, non-aversive closed arm, and open arms), as well as immobility time (indirect measure of sedative-motor effects) using the ANY-maze^®^ software (version 5.1, Stoelting). Immobility was calculated as the total time spent immobile during the training session. Learning and memory were assessed by quantifying the difference in the percentage of time spent in the aversive closed arm compared to the non-aversive closed arm during the training and test sessions, respectively. Anxiolytic-like effects were measured by the percentage of time spent in the open arms of the device during the training session (longer time spent in the open arms = decrease in anxiety-like behavior). Percent time spent in the open arms was calculated according to the equation: time spent in the open arms / total session time ^*^ 100. Percent time spent in the closed arms was calculated according to the equation: time spent in the arm of interest / total time spent in the closed arms ^*^ 100.

### 2.4. Conditioned place preference

The CPP apparatus consisted of two conditioning compartments of equal size (40 × 20 × 20 cm): compartment A, with black and white vertical lines on the walls and a black wooden floor, and compartment B, with black and white horizontal lines on the walls and a dark (red) smooth floor. The two main compartments were connected by a central compartment (40 × 10 × 15 cm) that was accessible by sliding doors. Test sessions were filmed, and the time spent in each compartment was measured using the ANY-maze^®^ software (version 5.1, Stoelting). The CPP procedure consisted of the following phases:

*Habituation (Day 1)*: Animals were placed in the center of the apparatus with free access to all compartments for 10 min. No treatments were administered.
*Pre-conditioning test (Day 2)*: Animals were placed in the center of the apparatus with free access to all compartments, and behavior was recorded for 15 min. No treatments were administered.
*Conditioning (Days 3–14)*: An unbiased design was used because animals showed no preference for either of the compartments in the pre-conditioning test. Therefore, animals were randomly assigned to an experimental group and to a “midazolam-paired compartment” in a counterbalanced manner. The conditioning sessions were performed during 12 consecutive days, during which the doors remained closed and animals were confined to one of the conditioning compartments. On odd days, animals received an intragastric administration of midazolam. On even days, animals received an intragastric administration of saline. Ten minutes after midazolam or saline administrations, animals were confined to the assigned drug- or saline-paired compartment for 10 min.
*Post-conditioning test (Day 15)*: Animals were placed in the center of the apparatus with free access to all compartments, and behavior was recorded for 15 min. No treatments were administered.

### 2.5. Two-bottle choice

Subjects were initially habituated to two 15 ml bottles of water for 3 days, followed by habituation to two 15 ml bottles containing 4% sucrose for another 3 days. Following this initial habituation phase, subjects had 24-h access to two 15 ml drinking bottles in their individual home cages, one containing 4% sucrose, and the other containing 4% sucrose plus midazolam (0.008, 0.016 or 0.032 mg/ml). All subjects were exposed to each concentration of midazolam for 14 days, and bottle sides were switched every 7 days. Consumption from each bottle was measured once every 24 h, at which time all subjects were weighed and bottles were refilled. In order to ensure data were not affected by liquid loss due to bottle leaks, for each cohort (Swiss vs. C57BL/6 cohorts) two bottles were left in an empty cage for 1 week, during which time liquid loss was measured and found to be <0.1 ml/day.

### 2.6. Experimental design

#### 2.6.1. Experiment 1. Evaluation of the anxiolytic-like, cognitive and rewarding effects of the benzodiazepine midazolam

Forty-eight Swiss male mice were randomly distributed into four groups and submitted to the elevated plus maze-discriminative avoidance task procedure, as described previously. On the training day, animals received an intragastric administration (gavage) of vehicle solution (*n* = 18) or midazolam at doses of 1 (MDZ 1, *n* = 12), 2 (MDZ 2, *n* = 18) or 5 (MDZ 5, *n* = 12) mg/kg. Ten minutes after administration, animals were placed individually in the center of the apparatus and had free access to all arms of the apparatus for 10 min. Twenty-four hours after the training session, all animals were submitted to a 3-min drug-free test session.

One week after the test day, the animals treated with midazolam during the discriminative avoidance task experiment were submitted to the CPP protocol, as described previously. Animals were maintained in the same midazolam groups, receiving the same dose of midazolam during the discriminative avoidance and the CPP protocols. Animals were submitted to the habituation and pre-conditioning test sessions. During the conditioning phase, animals received intragastric administration (gavage) of midazolam (1, 2 or 5 mg/kg, groups MDZ1, MDZ 2 and MDZ 5, respectively; *n* = 12 per group) on odd days and were confined to the drug-paired compartment for 10 minutes. On even days, all animals received intragastric administration (gavage) of saline and were confined to the opposite compartment for 10 min.

Twenty-four hours after the last day of the conditioning protocol, the post-conditioning test was performed, and the time spent in each of the main compartments was recorded. Expression of drug-induced CPP or conditioned place aversion was determined using the “score” measure (time spent in the drug-paired compartment minus time spent in the saline-paired compartment). A longer time spent in the compartment associated with the drug compared to the compartment paired with saline (positive score) was considered as indicative of the development of midazolam-induced CPP, while a negative score indicated midazolam-induced place aversion. A score of 0 indicates no preference.

#### 2.6.2. Experiment 2. Evaluation of midazolam choice behavior

Twenty-nine Swiss (outbred) male mice and 43 C57BL/6 (inbred) male mice were submitted to the habituation and TBC protocols, as previously described. Consumption of sucrose and midazolam plus sucrose solutions were averaged for the last 3 days of self-administration of each midazolam concentration (0.008, 0.016 or 0.032 mg/ml). Preference or aversion for the midazolam bottle over the sucrose only bottle was assessed for each midazolam concentration by calculating the consumption of the midazolam bottle / total consumption of the two bottles ^*^ 100.

### 2.7. Statistical analyses

The behavioral data from each experiment (Discriminative avoidance: % time spent on each arm of the device or immobility time; CPP: score; TBC: % preference) were analyzed using one- or two-way analysis of variance (ANOVA), with or without repeated measures (specific analyses described in the results section for each experiment). For all analyses, Bonferroni *t*-tests were used as the *post-hoc* test. In addition to the dependent measures listed above, three derived scores were calculated, including: (1) CPP score = time spent in the drug-paired compartment–time spent in the non-drug-paired compartment during the post-conditioning test session; (2) Learning score = time spent in the non-aversive closed arm–time spent in the aversive closed arm during the EPM training session; and (3) Memory score = time spent in non-aversive closed arm–time spent in the aversive closed arm during the EPM test session (24 h after training). The behavioral data from each experiment were analyzed using one- or two-way analysis of variance (ANOVA), with or without repeated measures (specific analyses indicated in the results section for each experiment). For all analyses, Bonferroni *t*-tests were used for multiple comparison tests. These analyses, as well as all graphical representations, were performed using the GraphPad Prism software (version 9).

Initial analyses of the data for the CPP studies revealed considerable variance for the CPP score, with distributions of scores predominantly positive (i.e., above zero, or no preference) but with negative scores (i.e., aversion) of relatively high magnitude. We proposed that mice showed either a significant preference or aversion to the midazolam-paired chamber, which represent diametrically opposed predictions for a drug reported consistently to have rewarding effects. Similarly, the TBC studies showed considerable variance for the percentage of preference for the midazolam-containing bottle, leading to a related prediction that mice either preferred or avoided consumption of midazolam. To test these possibilities, we conducted 2-step cluster analysis using CPP score and TBC preference measures. Two-step cluster analysis is a hybrid approach that first calculates a distance measure (centroids) to separate groups, followed by a probabilistic approach to choose an optimal subgroup (36, 37). Distance measures were determined by the log-likelihood criterion and cluster numbers were determined by the Schwarz Bayesian Criterion, with a default of 15 clusters iterations total.

Cohesion and separation of clusters was evaluated using the silhouette coefficient. Internal validity was evaluated further by comparing clusters using unpaired *t*-tests, Fisher's exact tests (categorical data), ANOVA and planned Bonferroni *t*-tests (to test for dose-associated effects), as well as conducting repeated clustering (*n* = 15) with newly randomized order of data for each analysis (cluster results can depend on order of data entered). External validation presented more difficulties, because of the lack of available data and analytic approaches providing construct validity associating either concurrent or mechanistic measures of midazolam reward. This study tested two hypotheses that addressed external validation: Midazolam reward is mediated by (1) reduction of anxiety (anxiolysis) and (2) associative learning and memory processes. To assess these hypotheses, correlation (Pearson r) and regression analysis were performed with CPP Score as a predictor of time in open arm (anxiolysis), learning and memory score. In addition, concepts of preference and aversion in CPP procedures are conceptually related to preference and avoidance in the TBC procedure, although as with our hypotheses, there are no available data to address these comparisons directly. Regardless, a general concordance between CPP and TBC with regards to number of clusters (i.e., preference vs. aversion/avoidance) would provide external validation. Cluster analyses were performed using IBM SPSS Statistics software (version 28). For all analyses, family-wise error rate (alpha) was constrained to *p* ≤ 0.05.

## 3. Results

### 3.1. Experiment 1. Evaluation of the anxiolytic-like, cognitive and rewarding effects of the benzodiazepine midazolam

#### 3.1.1. Elevated plus maze-discriminative avoidance task

**Figure 1:**
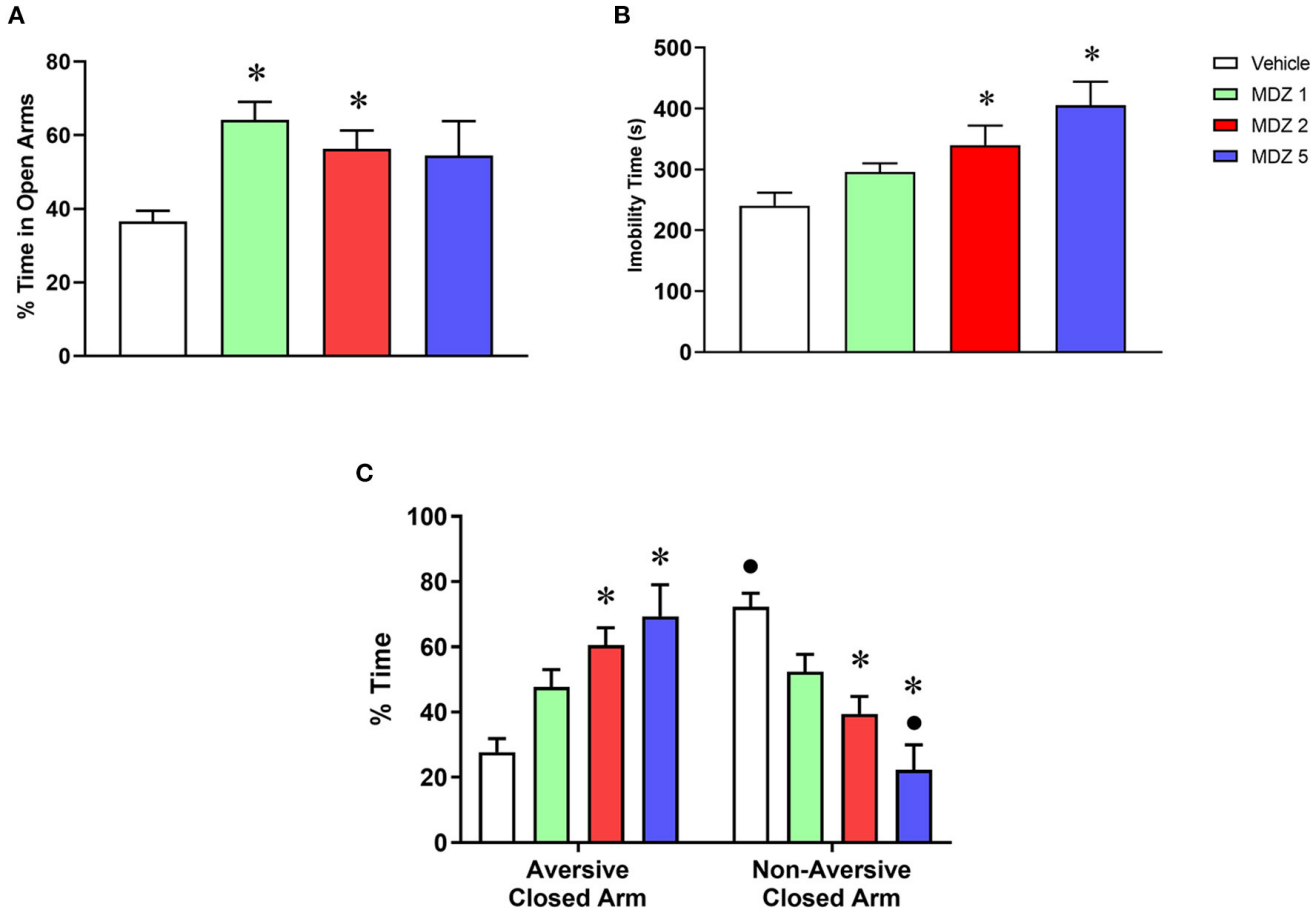
Results from the training session of the elevated plus maze-discriminative avoidance task following i.p. administration of vehicle (*n* = 18) or midazolam at the doses of 1 (MDZ 1, *n* = 12), 2 (MDZ 2, *n* = 18) or 5 (MDZ 5, *n* = 12) mg/kg. **(A)** Time spent in the open arms (anxiety-like behavior); **(B)** immobility time (sedative-motor effects); **(C)** time spent in the aversive vs. non-aversive closed arms (learning). Data are shown as mean ± SEM. *p < 0.05 compared to Vehicle within the same parameter; •*p* < 0.05 compared to time spent in the aversive closed arm within the same group.

Results from the training session of the discriminative avoidance experiment are illustrated in Figure 1, including analyses of anxiety-like (Figure 1A), sedative-motor (Figure 1B) and learning (Figure 1C) behaviors. One-way ANOVA of the % time spent in the open arms showed a statistically significant difference between groups [F_(3, 56)_ = 4.792; *p* < 0.01]. The two lowest doses of midazolam (1 and 2 mg/kg, *p* < 0.01 and *p* < 0.05, respectively, Bonferroni tests) significantly increased the % time spent in the open arms compared to vehicle. One-way ANOVA of immobility time also showed a statistically significant difference between groups [F_(3, 44)_ = 6.093; *p* < 0.01], with Bonferroni tests showing a significant increase in immobility time for the two highest doses of midazolam (2 and 5 mg/kg, *p* < 0.05 and *p* < 0.001, respectively) compared to vehicle. A two-way repeated measures ANOVA showed a significant interaction between compartment (aversive closed arm vs. non-aversive closed arm) and treatment (vehicle vs. midazolam) for the % time spent in the closed arms [F_(3, 55)_ = 12.52; *p* < 0.0001]. Bonferroni *post-hoc* analyses showed that only the vehicle group spent a significantly greater % time in the non-aversive closed arm compared to the aversive closed arm (*p* < 0.001), demonstrating that animals learned to avoid the aversive closed arm. No significant differences were observed in the % time spent in the closed arms for the groups treated with the lowest doses of midazolam (1 and 2 mg/kg), and animals treated with the highest dose of midazolam (5 mg/kg) spent a significantly lower % time in the non-aversive closed arm compared to the aversive closed arm (*p* < 0.01), suggesting that all doses of midazolam impaired learning. In agreement, animals treated with 2 and 5 mg/kg midazolam were also significantly different from the vehicle group for both % time spent in the closed aversive arm (*p* < 0.001 and *p* < 0.0001, respectively) and % time spent in the non-aversive closed arm (*p* < 0.001 and *p* < 0.0001, respectively).

**Figure 2:**
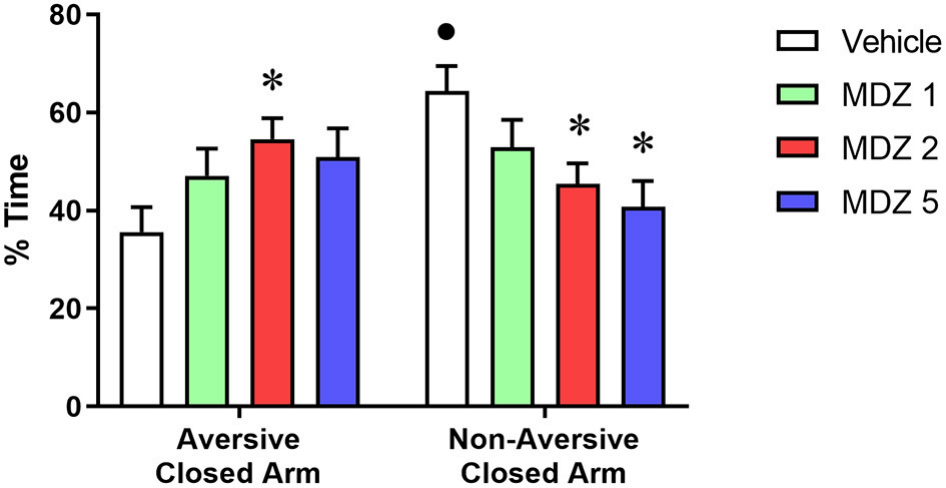
Results from the test session of the elevated plus maze-discriminative avoidance task 24-h after the training session, during which animals received i.p. administration of vehicle (*n* = 18) or midazolam at the doses of 1 (MDZ 1, *n* = 12), 2 (MDZ 2, *n* = 18) or 5 (MDZ 5, *n* = 12) mg/kg. No drugs were administered before the test session. Time spent in the aversive vs. non-aversive closed arms (memory retrieval). Data are shown as mean±SEM. *p < 0.05 compared to Vehicle within the same parameter; •*p* < 0.05 compared to time spent in the aversive closed arm within the same group.

Results from the memory measures during the test session of the discriminative avoidance experiment are illustrated in Figure 2. For the analysis of the % time spent in the closed arms, two-way repeated measures ANOVA showed a significant interaction between compartment (aversive closed arm vs. non-aversive closed arm) and treatment (vehicle vs. midazolam) [F_(3, 55)_ = 3.968; *p* < 0.05]. Only the vehicle group spent a significantly greater % time in the non-aversive closed arm compared to the aversive closed arm (*p* < 0.01), indicating that animals learned the association between aversive and non-aversive arms during the training session. No significant differences were observed between the % time spent in the aversive vs. non-aversive closed arms for midazolam-treated animals. Animals treated with midazolam spent a significantly higher % time in the aversive closed arm (*p* < 0.05 for 2 mg/kg) and a significantly lower % time in the non-aversive closed arm (*p* < 0.05 for 2 and 5 mg/kg) compared to the vehicle group.

#### 3.1.2. Conditioned place preference

**Figure 3:**
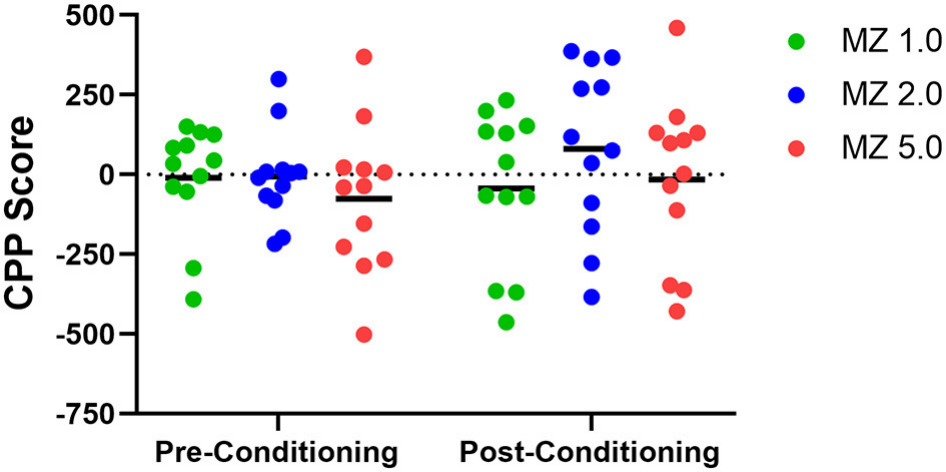
Conditioned place preference (CPP) and place aversion (CPA) in male Swiss mice (*n* = 12 per group) following midazolam doses (1.0, 2.0, and 5.0 mg/kg, i.g.). Data are CPP scores (time spent in drug-paired compartment–time spent in non-drug-paired compartment) after pre-exposure to the chambers (Pre-Conditioning) and after pairings with midazolam (Post-Conditioning). Individual points represent individual subjects, and horizontal bars represent mean values.

CPP results, measured as the difference in time spent in the midazolam- and saline-paired side (CPP score), are shown as a function of pre-conditioning and post-conditioning in Figure 3. During pre-conditioning, CPP scores for individual mice tended to aggregate near zero, with variability contributed by 2–4 mice at each dose condition. However, a more distributed set of CPP scores at each dose was observed in the post-conditioning tests. Two-way repeated measures ANOVA showed no significant differences of dose or conditioning phase [e.g., dose x conditioning phase interaction: F_(2, 33)_ = 0.593, *p* = 0.558].

**Figure 4:**
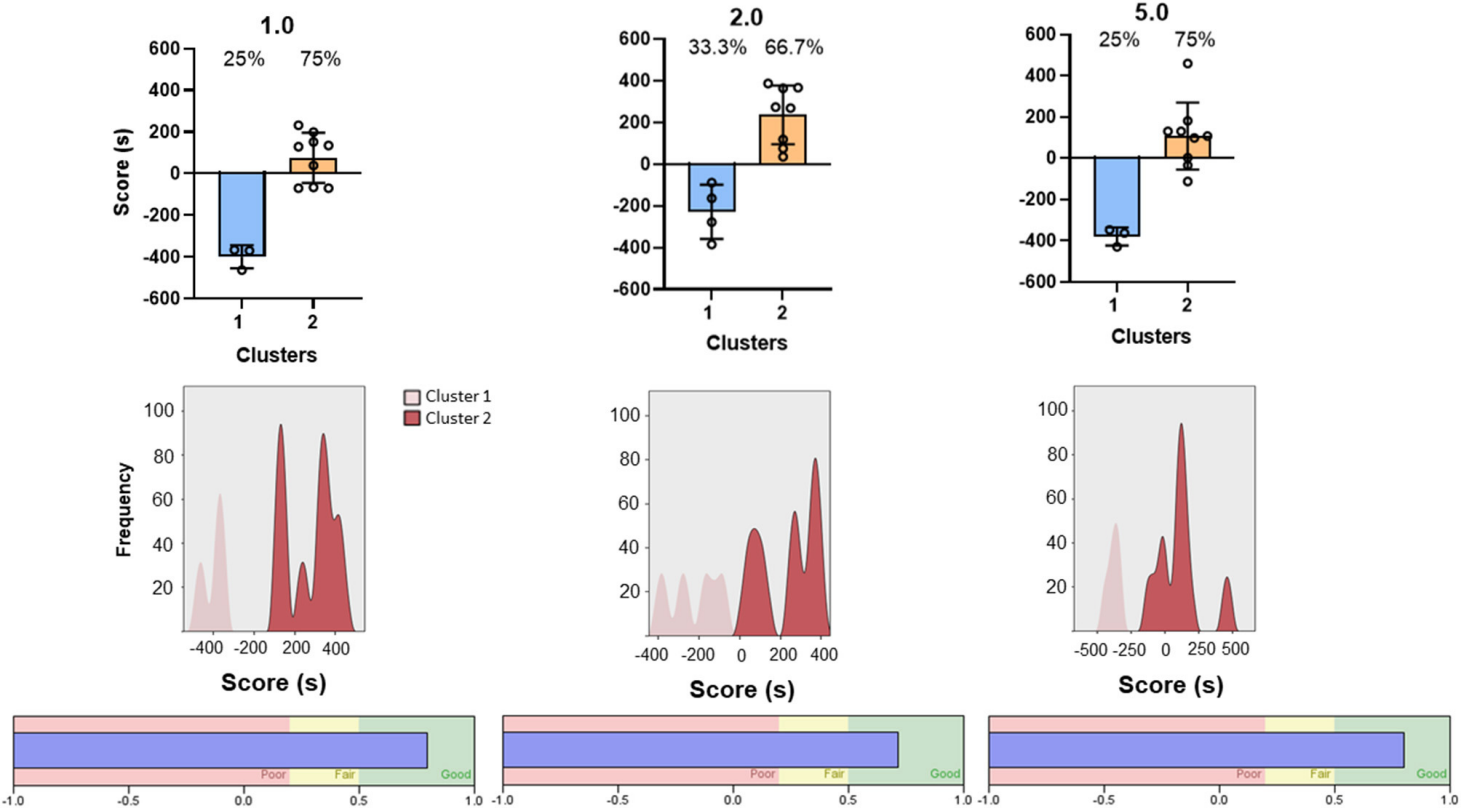
Two-step cluster analysis of midazolam-induced conditioned place preference in Swiss mice (*n* = 12 per dose). Each column represents analysis performed on each of three doses of midazolam, indicated at the top (1.0, 2.0, and 5.0 mg/kg, p.o.). **(Top)** panels are CPP scores (time spent in drug-paired compartment–time spent in non-drug-paired compartment) as a function of the 2 identified clusters (cluster 1–aversion, cluster 2–preference). Data are individual subjects, with bars representing mean values and error bars showing SEM values. Numbers in the panels represent percentages of mice in each cluster. **(Middle)** panels are frequency distributions (relative percentage) for each dose. **(Bottom)** panels show corresponding silhouette analysis for cohesion and separation of the clusters.

Because the CPP score represents a dichotomous variable, with positive numbers indicating preference and negative numbers indicating aversion, we explored the possibility of mice falling into distinct categories. Two-step clustering analysis was conducted separately for each dose of midazolam (1.0, 2.0, 5.0 mg/kg). Schwarz Bayesian Criterion (BIC) reached acceptable clustering with two centroids for all three doses. Figure 4 shows the results of this cluster analysis, with cluster 1 = aversion, i.e., negative numbers, and cluster 2 = preference, i.e., positive numbers. With some exceptions at 1.0 and 5.0 mg/kg, all CPP scores fell above or below zero, depending on the cluster. Based on the analysis, 66.7–75% of mice were grouped into the preference category (Figure 4, top panels), with frequency distributions showing the majority of subjects with CPP scores above zero and low degrees of overlap (Figure 4, middle panels). Silhouette analysis of cluster cohesion and separation showed index scores of 0.79 to 0.82, indicating “good” cluster quality (Figure 4, bottom panels). Repeated analyses with randomized data sets did not alter results. Importantly, we performed the same 2-step cluster analysis for the pre-conditioning data, based on the assumption that no clustering would be possible prior to any drug conditioning. Single clusters were obtained for the pre-conditioning phase for 2.0 and 5.0 mg/kg groups, and while two clusters were obtained for the 1.0 mg/kg group, the iterative process identified two outliers (CPP scores of −293 and −392) and resulted in a silhouette score categorized as “poor.” Interestingly, the two mice with the outlier scores remained at negative numbers for the post-conditioning test, indicating that they fell into the “aversion” cluster. Because these two subjects did not change to the “preference” cluster (which would result in a substantial change in CPP scores) and this pattern was not evident at the other two doses, we did not exclude the mice from any of the analyses.

**Figure 5:**
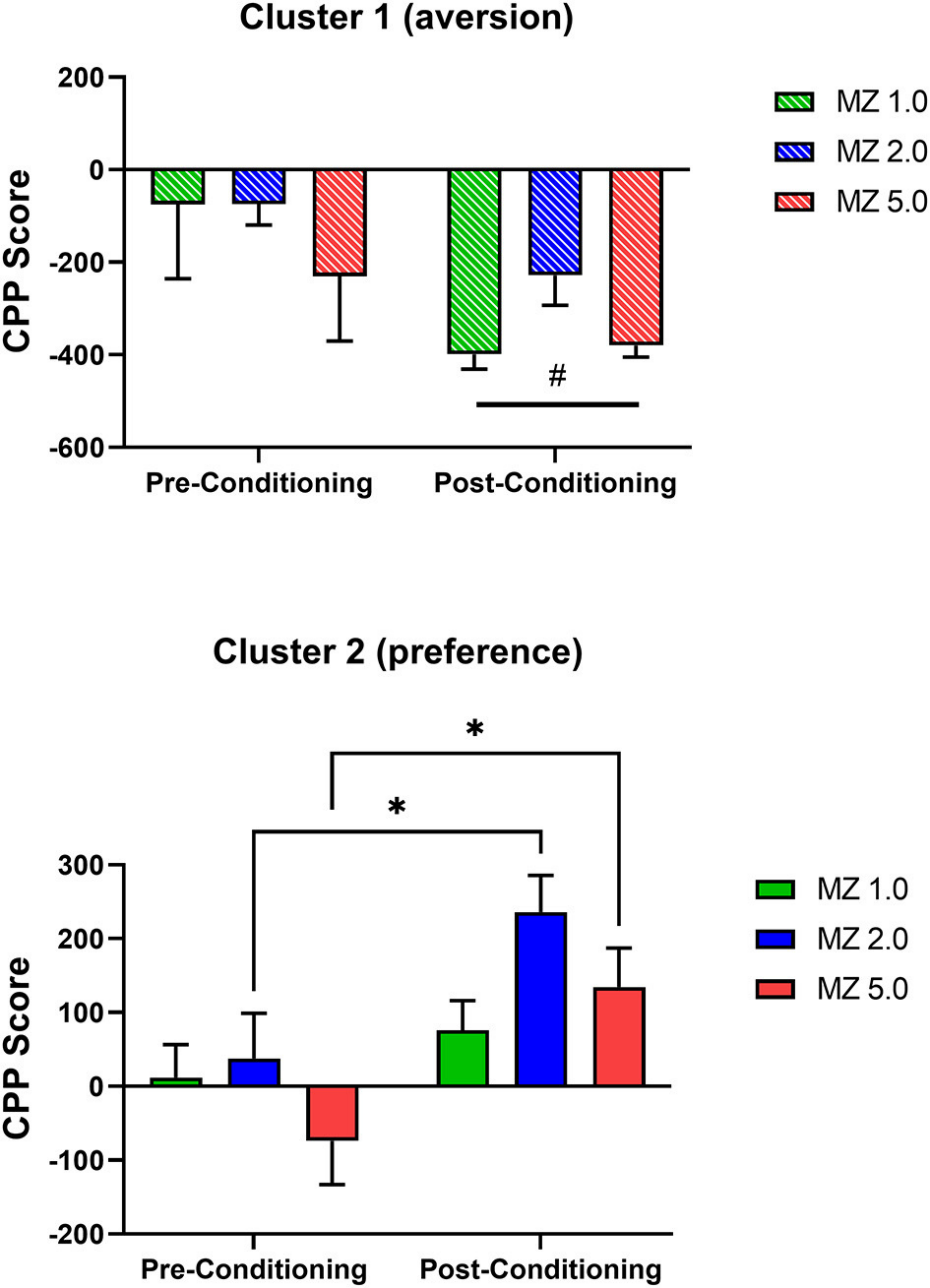
Conditioned place preference (CPP) results with midazolam (MZ) per clusters, determined by 2-step cluster analysis, in male Swiss mice. Data are expressed as mean +/- SEM CPP Score (time spent in drug-paired compartment–time spent in non-drug-paired compartment) for *N* = 36 mice total. Results are from pre-conditioning tests performed prior to midazolam–saline pairings and post-conditioning tests conducted after training sessions. The **(Top)** panel shows results from cluster 1 (aversion); note that #*p* < 0.05, main effect of conditioning phase, ANOVA. The **(Bottom)** panel shows results from cluster 2 (preference); note that *p < 0.05, Bonferroni *t*-tests.

We performed additional internal validation tests that also provided information on dose-dependency (Figure 5). For these analyses, the clusters were analyzed with separate repeated measures ANOVAs. For cluster 1 (aversion; Figure 5 top panel), the ANOVA demonstrated no effects of dose [dose main effect: F_(2, 7)_ = 1.469, *p* = 0.293; dose x conditioning phase interaction: F_(2, 7)_ = 0.677, *p* = 0.539] but a significant main effect of conditioning phase [F_(1, 7)_ = 9.296, *p* = 0.019], indicating that a significant aversion occurred, irrespective of dose. To test specifically for dose-related effects, Bonferroni *t*-tests were conducted within the doses pre- and post-conditioning; and no significant differences were evident (p's > 0.05).

For cluster 2 (preference, Figure 5 bottom panel), the repeated measures ANOVA showed no significant effects related to dose [dose main effect: F_(2, 23)_ = 2.118, *p* = 0.143; dose x conditioning phase interaction: F_(2, 23)_ = 0.672, *p* = 0.520] but a significant main effect of conditioning phase [F_(1, 22)_ = 7.866, *p* = 0.010]. Planned Bonferroni *t*-tests showed that an increase in time spent in the drug-paired side occurred for the 2.0 and 5.0 mg/kg doses of midazolam (adjusted *p* = 0.031 and 0.023, respectively), indicating significant dose-dependent CPP with midazolam for cluster 2.

**Figure 6:**
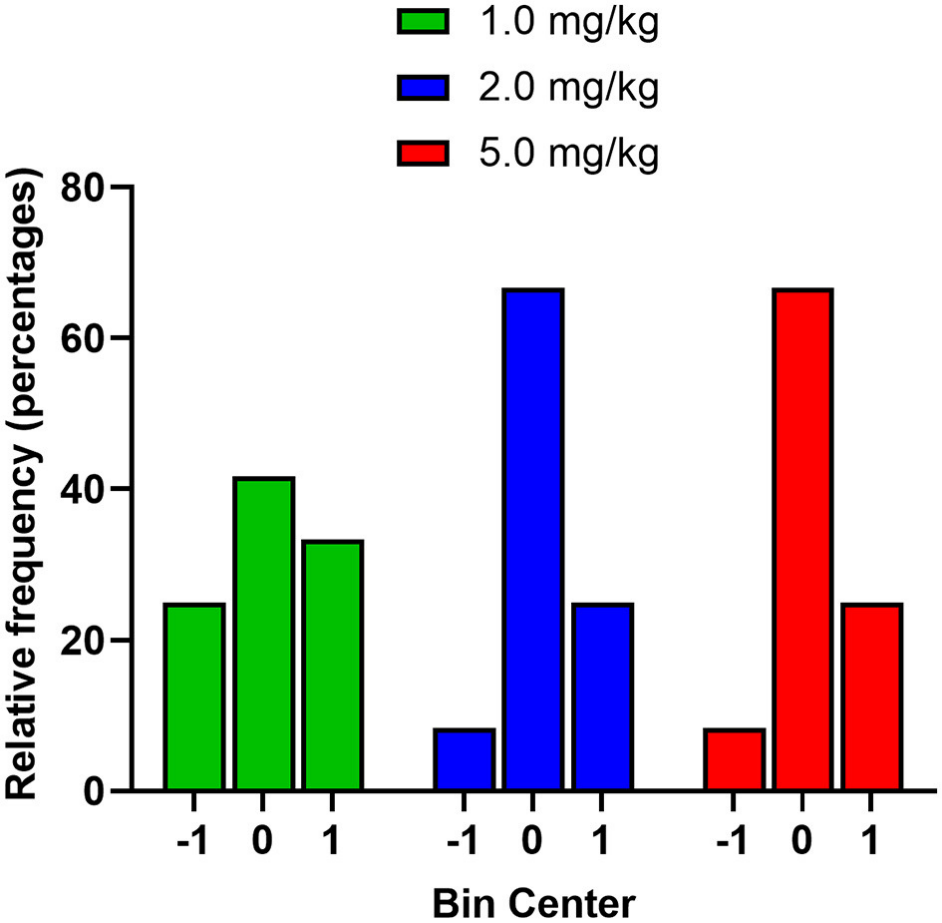
Frequency distribution of male Swiss mice based on change from pre- to post-conditioning in the CPP tests (*n* = 12 per dose). Mice were coded with three numbers according to the following categories: −1.0, mice showing positive scores in pre-conditioning and negative in post-conditioning; 0, mice that stayed either positive or negative in pre-conditioning and post-conditioning; +1.0, mice showing negative scores in pre-conditioning and positive scores in post-conditioning.

Based on the cluster analysis, the majority of mice showed preference for the midazolam-paired compartment; an effect off-set by mice showing aversion. It was notable that there was variance in the pre-conditioning phase, with one group even displaying 2 clusters, raising the likelihood that the mice demonstrated preference or aversion in the absence of drug conditioning. To evaluate the nature of change in CPP score from pre- to post-conditioning, we first coded mice with three numbers according to the following categories: −1.0, mice showing positive scores in pre-conditioning and negative in post-conditioning; 0, mice that stayed either positive or negative in pre-conditioning and post-conditioning; +1.0, mice showing negative scores in pre-conditioning and positive scores in post-conditioning. A frequency histogram was plotted (Figure 6), showing that for each dose, the majority of mice did not change from pre- to post-conditioning, i.e., if they showed a negative pre-conditioning score, they showed a negative post-conditioning score. The next highest frequency was the mice showing a change from negative to positive CPP scores after conditioning, with a smaller number of mice (25% for all three doses) demonstrating a shift from positive to negative CPP scores.

#### 3.1.3. Comparison of parameters from conditioned place preference and elevated plus maze-discriminated avoidance tasks

In order to obtain information regarding potential behavioral mechanisms underlying midazolam-induced conditioned place preference, we conducted additional analyses to determine the extent to which CPP score in mice showing place preference could predict effects in the EPM (time spent in the open arm, i.e., anxiolytic-like effects; learning and memory scores). Correlation matrices (Pearson r) are shown in Table 1 for the 4 measures, conducted within each dose of midazolam. As evident from the table, no correlations were significant with CPP score, although significant positive correlations were obtained for time in open arms for memory score at 1.0 mg/kg midazolam and learning score at 5.0 mg/kg of midazolam.

**Table 1:** Correlation matrices (Pearson's r values) for Swiss mice in the conditioned place preference and elevated plus maze-discriminative avoidance tasks (mice from cluster 2 only).

**1.0 mg/kg Midazolam**	**CPP score**	**Time in open arms**	**Learning score**	**Memory score**
CPP score	1.000	0.392	0.303	0.030
Time in open arms	0.392	1.000	0.060	0.745^*^
Learning score	0.303	0.060	1.000	0.083
Memory score	0.030	0.745^*^	0.083	1.000
**2.0 mg/kg Midazolam**	**CPP score**	**Time in open arms**	**Learning score**	**Memory score**
CPP score	1.000	0.105	−0.211	0.456
Time in open arms	0.105	1.000	0.529	0.383
Learning score	−0.211	0.529	1.000	0.150
Memory score	0.456	0.383	0.150	1.000
**5.0 mg/kg Midazolam**	**CPP score**	**Time in open arms**	**Learning score**	**Memory score**
CPP score	1.000	0.451	0.287	−0.550
Time in open arms	0.451	1.000	0.789^*^	−0.228
Learning score	0.287	0.789^*^	1.000	−0.157
Memory score	−0.550	−0.228	−0.157	1.000

* p < 0.05, Pearson's r correlation.

CPP score (CPP test) = time spent in drug-paired compartment–time spent in non-drug-paired compartment.

Time Open Arms (EPM) = total time spent in the open arms during training session.

Learning score (EPM training session) = time spent in non-aversive closed arm–time spent in the aversive closed arm.

Memory score (EPM test session) = time spent in non-aversive closed arm–time spent in the aversive closed arm.

To test specifically if CPP score was a reliable predictor of EPM-DA parameters, individual linear regression analyses were conducted for the cluster 2 (preference) mice (Table 2). In every case, the regression parameter values were not significantly different from zero, with relatively low goodness-of-fit values (R2). Therefore, no evidence to support the hypotheses that CPP reflects anxiolytic or learning and memory associated processes were obtained for this data set.

**Table 2:** Linear regression analysis for Conditioned Place Preference as a predictor of Elevated Plus Maze-Discriminative Avoidance task performance (*n* = 12 Swiss mice per dose, from cluster 2 only).

	**Regression parameter value (SEM)**		
**1.0 mg/kg Midazolam**	**Time in open arms**	**Learning Score**	**Memory score**
Y-intercept	279.93 (31.60)	−17.34 (37.31)	−3.06 (12.78)
Slope	0.26 (0.23)	0.23 (0.27)	0.007 (0.094)
R^2^	0.15	0.09	0.01
**2.0 mg/kg Midazolam**			
Y-intercept	242.30 (72.49)	−38.92 (72.27)	−64.84 (40.09)
Slope	0.07 (0.27)	−0.14 (0.27)	0.19 (0.15)
R^2^	0.01	0.21	0.04
**5.0 mg/kg Midazolam**			
Y-intercept	194.19 (66.85)	−215.41 (101.63)	−3.75 (11.95)
Slope	0.48 (0.36)	0.43 (0.55)	−0.11 (0.06)
R^2^	0.20	0.08	0.30

Dependent variable was CPP score (CPP test) = time spent in drug-paired compartment–time spent in non-drug-paired compartment.

Time Open Arms (EPM) = total time spent in the open arms during training session.

Learning score (EPM training session) = time spent in non-aversive closed arm–time spent in the aversive closed arm.

Memory score (EPM test session) = time spent in non-aversive closed arm–time spent in the aversive closed arm.

### 3.2. Experiment 2. Evaluation of midazolam choice behavior

**Figure 7:**
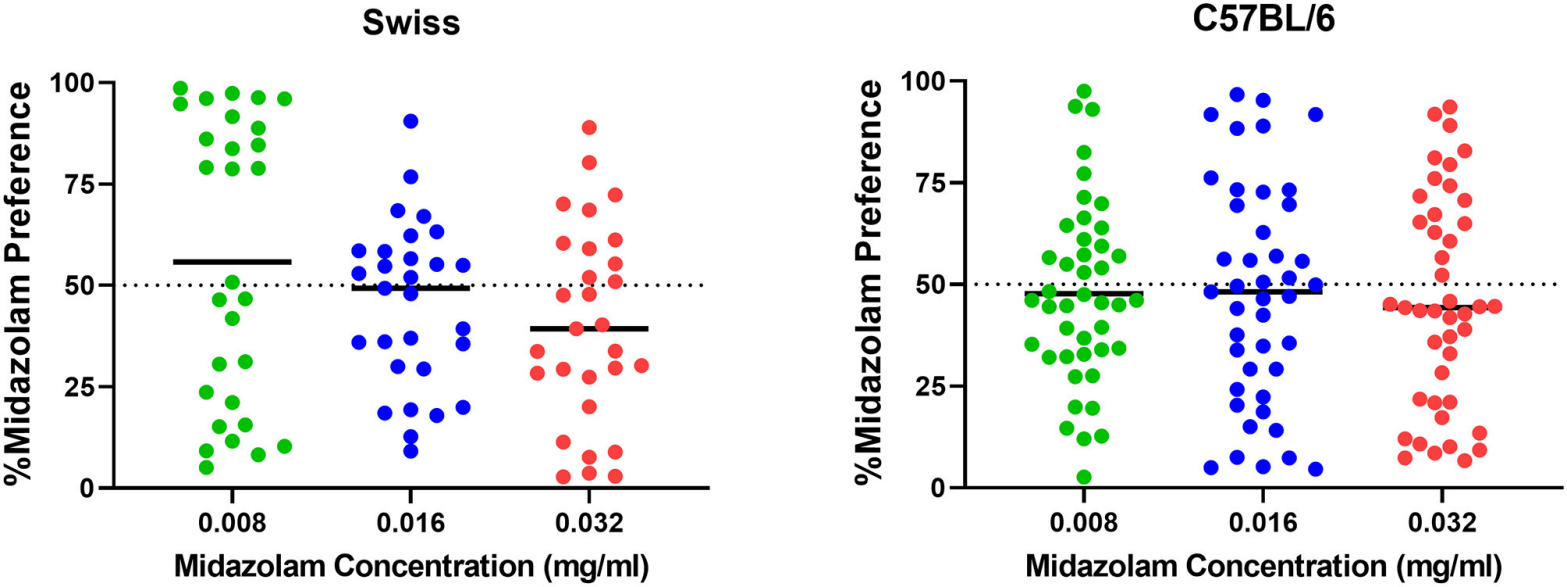
Two-bottle choice results following midazolam concentrations (0.008, 0.016, 0.032 mg/ml) in male Swiss mice (*N* = 36) and male C57BL/6 mice (*N* = 43). Data are percentage of midazolam preference, calculated as the volume consumed from the midazolam bottle/total volume consumed, multiplied by 100. Note that values above 50% indicate preference for midazolam, whereas values below 50% indicate avoidance of the midazolam bottle. Individual points represent individual subjects, and horizontal bars represent mean values.

**Figure 8:**
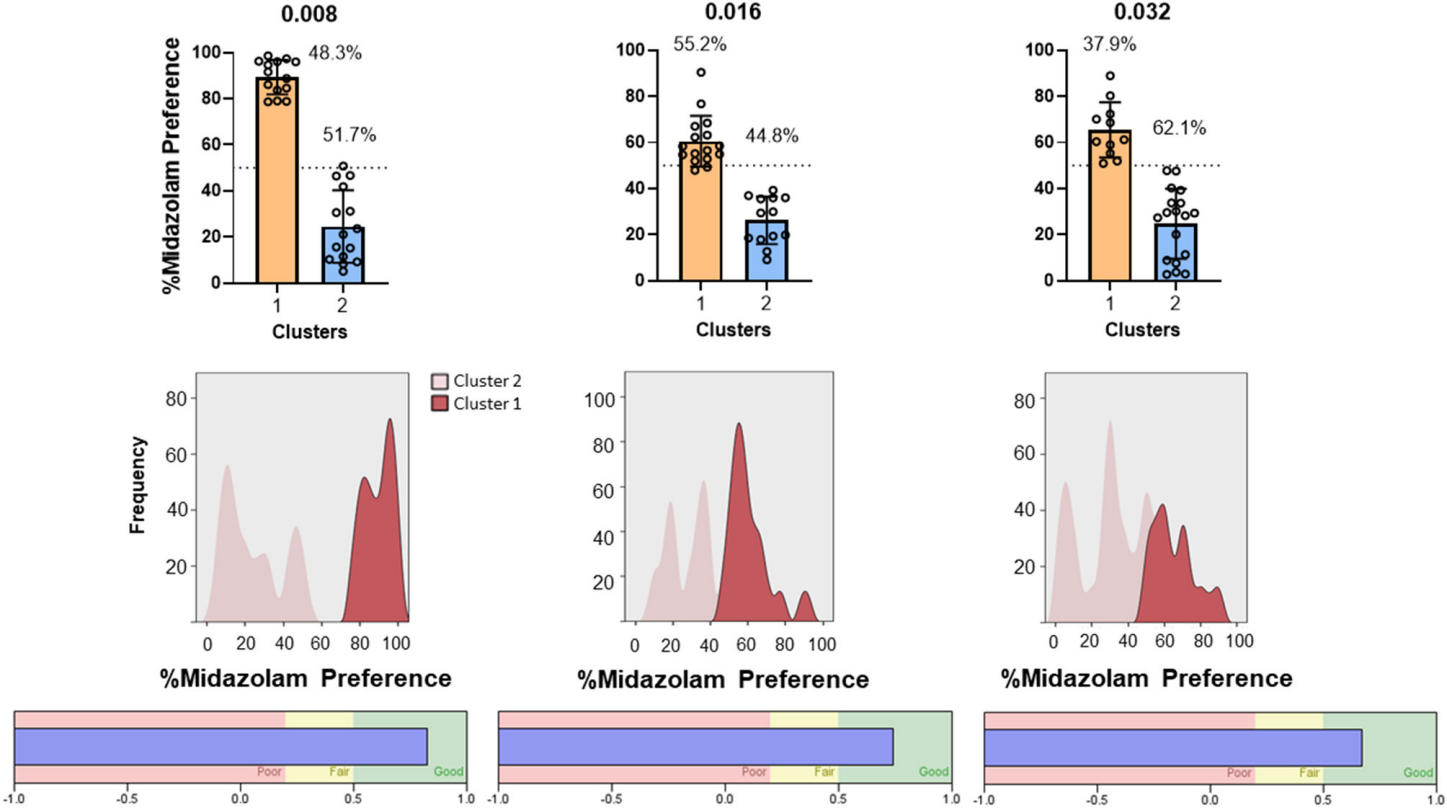
Two-step cluster analysis of midazolam preference in the two-bottle choice procedure in male Swiss mice (*N* = 36 mice total). Each column represents analysis performed on each of three concentrations of midazolam, indicated at the top (0.008, 0.016, 0.032 mg/ml). **(Top)** panels are percent midazolam preference (the volume consumed from the midazolam bottle/total volume consumed, multiplied by 100) as a function of the 2 identified clusters (cluster 1–aversion, cluster 2–preference). Data are individual subjects, with bars representing mean values and error bars showing SEM values. Numbers in the panels represent percentages of mice in each cluster. **(Middle)** panels are frequency distributions (relative percentage) for each dose. **(Bottom)** panels show corresponding silhouette analysis for cohesion and separation of the clusters.

Results from TBC tests with both Swiss and C57BL/6 mice cohorts are shown in Figure 7. For Swiss mice (Figure 7, left panel), ANOVA revealed a significant effect of concentration [F_(2, 56)_ = 4.383, *p* = 0.030]; however, no multiple comparisons were significant (p's > 0.05, Bonferroni *t*-tests). For C57BL/6 mice, the overall ANOVA was not significant [F_(2, 84)_ = 0.099, *p* = 0.899]. However, as with CPP in Swiss mice, midazolam preference was highly variable, with mice showing both preferences (above 50%) and avoidance (below 50%). We used 2-step cluster analyses to evaluate the extent to which mice in these studies could be parsed into those that preferred midazolam above 50% levels and those that avoided consuming the drug. For Swiss mice, all three concentrations resulted in 2 clusters (Figure 8). In general, the distribution of mice into the two clusters was approximately equal, with very little overlap among clusters (Figure 8, top and middle panels). The differences in percent midazolam preference between clusters was confirmed by unpaired *t*-tests [0.008 mg/ml: t_(27)_ = 14.01, *p* < 0.0001; 0.016 mg/ml: t_(27)_ = 8.596, *p* < 0.0001; t_(27)_ = 7.556, *p* < 0.0001]. For all three doses, the silhouette scores were in the “good” range (i.e., >0.5; Figure 8, bottom panels).

**Figure 9:**
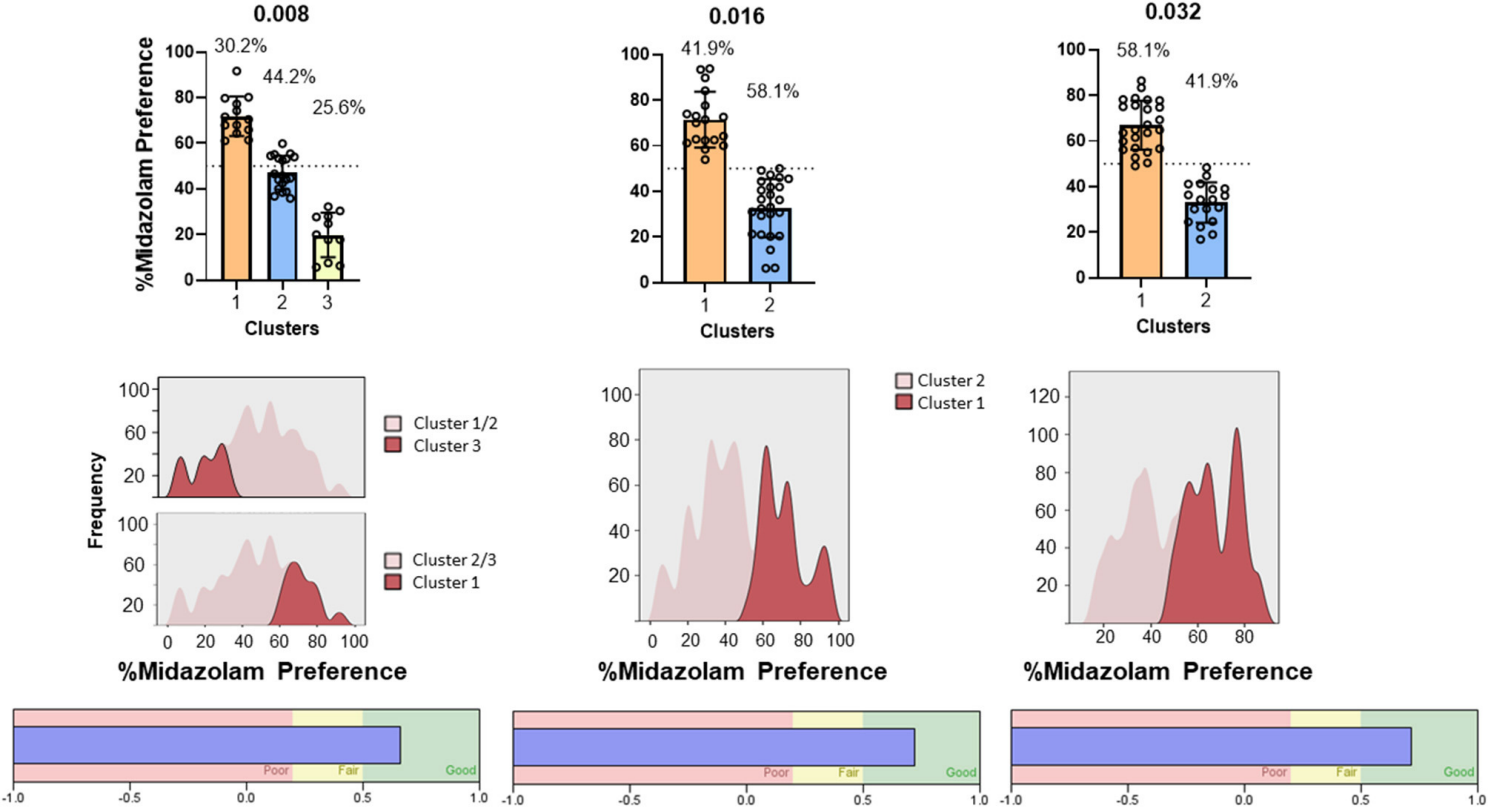
Two-step cluster analysis of midazolam preference in the two-bottle choice procedure in male C57BL/6 mice (*N* = 43 mice total). Each column represents analysis performed on each of three concentrations of midazolam, indicated at the top (0.008, 0.016, 0.032 mg/ml). Other details as in Figure 8.

For C57BL/6 mice, two of the concentrations resulted in similar clustering to the Swiss mice (0.016 and 0.032 mg/ml; Figure 9, top panels). Two-step cluster analysis of the lowest concentration, however, resulted in 3 clusters, with a cluster predominantly above equal preference (cluster 1, mean = 71.9 %, “preference”), a cluster near equal preference (cluster 2, mean = 44.7%, “indifference”), and a cluster well below equal preference (cluster 3, mean = 17.7%, “avoidance”). ANOVA performed on these data was significant [F_(2, 40)_ = 113.4, *p* < 0.0001] and multiple comparisons confirmed that all clusters were significantly different from one another (Bonferroni tests, adjusted p's < 0.0001). As with the Swiss mice, the two higher concentrations resulted in clustering into two groups that were nearly evenly distributed: 0.016 mg/ml, cluster 1 (preference) = 41.9%, cluster 2 (avoidance) = 58.1%; 0.032 mg/ml, cluster 1 (preference) = 58.1%, cluster 2 (avoidance) = 41.9%. Statistical comparisons verified the differences: 0.016 mg/ml, t_(41)_ = 10.04, *p* < 0.0001; 0.032 mg/ml, t_(41)_ = 10.98, *p* < 0.0001. In addition, silhouette scores for all three analyses fell between 0.5 and 1.0, indicating good cohesion and separation of clusters (Figure 9, bottom panels).

**Figure 10:**
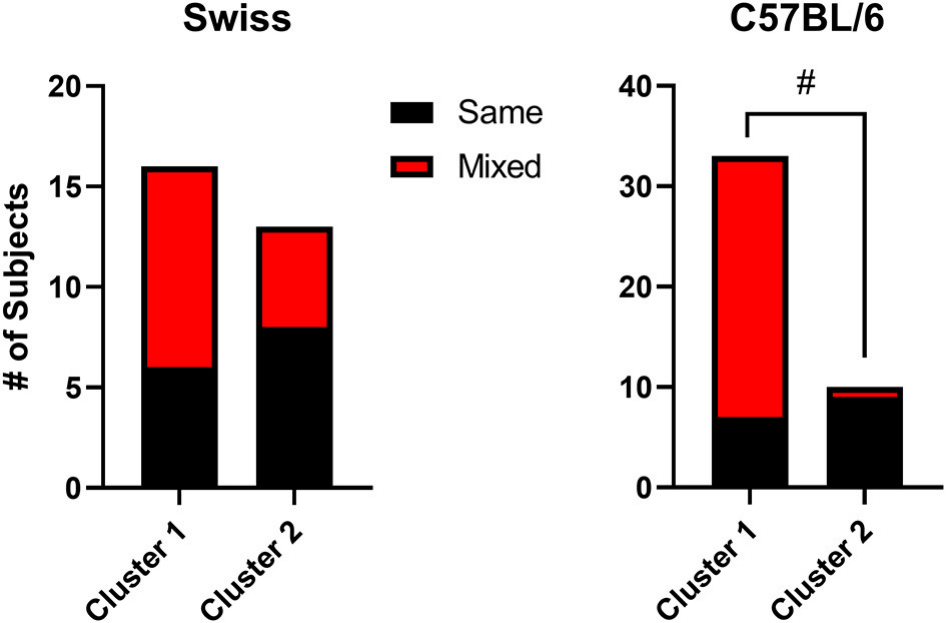
Stacked bar charts showing the number of mice coded as “same” (indicating a mouse for which all three concentrations were either above 50% preference or ≤50% preference) or “mixed” (indicating a mouse for which at least one concentration differed from the other concentrations). Data are separated by cluster 1 (preference) and cluster 2 (avoidance) for the two strains (Swiss and C57BL/6 male mice). Note that #*p* < 0.05, Fisher's exact test.

A noteworthy characteristic of the TBC data is the observation that different mice were in different clusters across the concentrations. This is expected, because many mice showed mixtures of preference and avoidance depending on the concentration, as is a fundamental characteristic of self-administration data over all drug classes and most procedures. To quantify this phenomenon, we coded each mouse as “same” of “mixed” effects. “Same” indicated a mouse for which all three concentrations were either above 50% preference or ≤50% preference. “Mixed” indicated a mouse for which at least one concentration differed from the other concentrations. For example, a mouse with preference above 50% preference for 0.016 mg/ml but below 50% preference for the other two concentrations was coded as “mixed.” For the two strains, we compared the two clusters by conducted Fisher's exact tests. As shown in Figure 10, for Swiss mice, both same and mixed categories were observed about equally and did not differ between the clusters (Fisher's exact test, *p* > 0.05). Interestingly, for C57BL/6 mice, cluster 1 (preference) mice were predominantly in the mixed category, whereas cluster 2 (avoidance) mice were infrequently coded as mixed (Fisher's exact test, *p* = 0.0002). This analysis indicates that for Swiss TBC results, effects dependent on dose accounted for half of the subjects in both clusters, whereas with C57BL/6 mice, effects were dependent on dose predominantly for the mice showing preference for the midazolam solutions.

## 4. Discussion

Despite the clear and growing concern over benzodiazepine misuse, investigating the abuse-related effects of these drugs has not been as straightforward as researchers might expect. The pre-clinical literature on the effects of benzodiazepines in animal models has been filled with contradictory findings, and establishing models to investigate benzodiazepine reward and reinforcement has been a challenge. Specifically, studies have shown opposite effects for benzodiazepine self-administration (5–9), benzodiazepine-induced CPP (10–14) and changes in brain dopamine levels induced by benzodiazepines (15–20).

In the present study, assessment of midazolam reward *via* the CPP procedure and choice *via* the TBC procedure showed notable variability, with evidence that mice developed CPPs or conditioned place aversions (CPAs) with midazolam exposure and, similarly, preferred or avoided midazolam in the TBC model. We evaluated the extent to which mice could be divided into broadly different categories, i.e., midazolam-preferring vs. midazolam non-preferring, using 2-step cluster analysis. This approach was used because it does not require a priori choice of number of possible clusters, opening up the possibility for identification of other sub-groups (e.g., mice demonstrating indifference to the midazolam solutions). Regarding the CPP data, midazolam data were well-fitted to 2 separate clusters, albeit with the majority of mice showing preference (75%). When analyzed separately, the 2.0 and 5.0 mg/kg doses engendered significant preference in the preference cluster, whereas the aversion cluster generally showed robust aversions with no dose-related effects. The majority of mice demonstrated CPP by an increase in time spent in the midazolam-paired compartment, mostly by increasing time spent in the particular chamber vs. shifting preference from one chamber to another. This latter observation suggests that mice already showing an aversion to a drug-paired chamber may not be likely to change to a preference, however, the mice in the aversion cluster mostly showed increased time in the non-drug-paired side instead of no change from pre-conditioning tests.

The distinct clusters observed for the CPP experiment allowed us to assess the relationship between reward and other characteristic effects of benzodiazepines. In this regard, midazolam had anxiolytic-like effects in mice, increasing the time spent in the open arms of the modified EPM apparatus, consistent with previous studies (38, 39). To investigate the relationship between the anxiolytic-like and rewarding effects of midazolam, we conducted correlational analysis as well as regressed CPP scores vs. time spent in the open arms of the EPM. These analyses showed no relationship between midazolam reward and this measure of anxiolytic-like effects, suggesting that the emergence of anxiolytic-like effects is not sufficient to guarantee the expression of rewarding effects. Interestingly, strong positive correlations were shown for learning and memory scores vs. time in the open arms, suggesting that a stronger anxiolytic-like effect was associated with a higher degree of learning and memory impairment. In fact, midazolam reducing the aversiveness of the open arm may play a key role in any learning/memory impairment associated with this particular task.

The finding that midazolam impaired learning and memory of a discriminative avoidance task is consistent with previous pre-clinical studies with midazolam (28) and other benzodiazepine-type drugs (24, 29, 40). Because the CPP model relies on associative learning, we also investigated a potential correlation between the rewarding (CPP) and cognitive-impairing (discriminative avoidance task) effects of midazolam. As with anxiolytic-like measures, we found no significant relationships between these two measures, suggesting that the rewarding and aversive effects of midazolam emerged despite significant learning deficits induced by this drug.

In addition to testing the hypotheses that midazolam reward is associated with its anxiolytic and cognitive-impairing effects, these comparisons potentially provided tests of external validity for the 2-step cluster approach. Clearly these findings did not provide external support for the clustering, with lack of a relationship between CPP and cognitive effects perhaps the most perplexing. However, it is critical to note that the effects of midazolam in the learning and memory components of the discriminative avoidance task were to impair these processes, whereas CPP and CPA involve forming associative pairings. Moreover, learning to avoid an open arm may represent a form of fear conditioning, as opposed to reward learning represented by CPP, which was the result of 75% of the mice, and while neural circuits mediating aversive and reward learning may overlap, there likely are distinct functional differences [e.g., (41)]. Regarding anxiolysis, the hypothesis that the expression of reward may reflect reductions in anxiety is based primary on self-report data from human subjects identifying motives for taking benzodiazepines [e.g., (4)], rather than data from laboratory animal studies. Collectively, these observations do not provide external validity for the clustering but also are insufficient to discount the clustering approach, given that anxiolysis and learning/memory were components of hypothesis testing and not empirical conclusions *per se*.

External validation of mice being categorized as midazolam-preferring vs. midazolam-averse comes primarily from the TBC experiments. Two-step cluster analysis demonstrated that two different strains of mice overall fell into two clusters identified as midazolam-preferring or midazolam-avoiding groups, with only one exception being the lowest concentration of midazolam tested in C57BL/6 mice, which resulted in an additional (third) cluster characterized as indifference (i.e., equal distribution of drinking from midazolam + sucrose and sucrose alone bottles). Both midazolam preference and avoidance were concentration-dependent in a subset of mice, with some showing preference at some concentrations but avoidance at others. However, there was a trend for this pattern to occur more frequently in the midazolam-preferring Swiss mice and a statistically significant difference between midazolam-preferring and midazolam-avoiding C57BL/6 mice, suggesting that mice in the midazolam-avoiding groups tended to only show avoidance regardless of the concentration of drug.

Wild type laboratory mice can be divided into two main genetic categories: inbred and outbred (42). Inbred mice, such as the C57BL/6 mouse strain, are genetically homogeneous, and there is little genetic variation within this strain, which can reduce experimental variability and allow for the evaluation of genetic influences on specific behavioral phenomena. Outbred mice, such as the Swiss mouse strain, are bred specifically to maximize genetic diversity and heterozygosity within a population and, in theory, there are no two genetically identical outbred subjects. Therefore, the use of genetically heterogeneous and homogeneous strains allowed us to assess whether genetic factors could influence the expression of midazolam preference vs. avoidance. Our findings showed that both inbred and outbred mice demonstrated a strikingly similar pattern of preference and avoidance in the TBC experiments, even with the two TBC studies conducted at separate facilities. These studies ruled out a potential influence of genetic factors in our findings, raising the possibility that midazolam preference vs. avoidance groupings may develop in mice due to epigenetic factors.

The present findings corroborate a recent study in non-human primates showing that only half of the subjects self-administered the benzodiazepine alprazolam intravenously, although that study was conducted in rhesus monkeys with a history of opioid self-administration (43). These findings are also in agreement with a choice study in humans showing that, while diazepam was always preferred over placebo, placebo was preferred over oxazepam in nearly 22% of choice tests by recreational benzodiazepine users (44). The mechanisms underlying these contrasting findings within a study remain unknown. However, the unique pharmacokinetic properties of midazolam and other benzodiazepines may have contributed to these results. Due to its pharmacokinetic and pharmacodynamic properties, midazolam induces hysteresis, which results in a delay between the peak drug serum concentrations and the peak drug behavioral effects (45). Hysteresis indicates that the relationship between drug concentration vs. drug effects is not a straightforward, direct relationship, but may have an inherent delay and imbalance, which may be a result of active metabolites, or a consequence of changes in pharmacodynamic properties (45). Importantly, studies have shown that hysteresis influences benzodiazepine self-administration in rats (46). Of note, hysteresis also has been reported for both alprazolam (47) and oxazepam (48). Although further studies are needed to understand how this specific effect could affect some animals but not others, these pharmacokinetic and pharmacodynamic mechanisms may have influenced our findings.

Overall, our findings show that midazolam preference is a multifactorial behavior, and is not dependent solely on the emergence of anxiolytic-like effects, or on genetic factors (inbred vs. outbred animals). Also, the rewarding effects of midazolam in the CPP model emerged even at doses that induced significant learning deficits in mice. The protocols established in the present study can be used in future research to evaluate the neuropharmacological mechanisms involved in the different behavioral effects of benzodiazepine drugs within the same group of animals. Of note, important limitations of our study include the lack of sex differences investigation, with the possibility that different results would have been obtained for female mice. Also, the sample size in our CPP studies limited some of our analyses, and future studies should consider including multiple cohorts of animals to increase sample size in order to better capture benzodiazepine-induced CPP vs. CPA in mice. Regardless, our data emphasize the importance of considering interindividual variability within a sample, and suggest that variability may be an inherent phenomenon to the study of the abuse-related behavioral effects of benzodiazepines. Embracing variability may provide new avenues of study and a better understanding on how and why benzodiazepine drugs are abused.

## Data availability statement

The raw data supporting the conclusions of this article will be made available by the authors, without undue reservation.

## Ethics statement

This animal study was reviewed and approved by the Institutional Animal Care and Use Committees of UESC (protocol #006/2017) and UMMC (protocol #1395).

## Author contributions

AO-L, EM, JR, and LB were responsible for the study concept and design. CJ-F, MF, BD, YS, NK, TB-S, NJ, IR, and LM contributed to data acquisition. CJ-F, EM-M, AO-L, EM, JR, and LB assisted with data analysis and interpretation of findings. LB and JR drafted the manuscript. All authors agree to be accountable for all aspects of the work, provided critical revision of the manuscript for important intellectual content, and approved the final version for publication.
